# scanPAV: a pipeline for extracting presence–absence variations in genome pairs

**DOI:** 10.1093/bioinformatics/bty189

**Published:** 2018-03-28

**Authors:** Francesca Giordano, Maximilian R Stammnitz, Elizabeth P Murchison, Zemin Ning

**Affiliations:** 1 The Wellcome Sanger Institute, Wellcome Genome Campus, Hinxton; 2 Transmissible Cancer Group, Department of Veterinary Medicine, University of Cambridge, Cambridge, UK

## Abstract

**Motivation:**

The recent technological advances in genome sequencing techniques have resulted in an exponential increase in the number of sequenced human and non-human genomes. The ever increasing number of assemblies generated by novel *de novo* pipelines and strategies demands the development of new software to evaluate assembly quality and completeness. One way to determine the completeness of an assembly is by detecting its Presence–Absence variations (PAV) with respect to a reference, where PAVs between two assemblies are defined as the sequences present in one assembly but entirely missing in the other one. Beyond assembly error or technology bias, PAVs can also reveal real genome polymorphism, consequence of species or individual evolution, or horizontal transfer from viruses and bacteria.

**Results:**

We present scanPAV, a pipeline for pairwise assembly comparison to identify and extract sequences present in one assembly but not the other. In this note, we use the GRCh38 reference assembly to assess the completeness of six human genome assemblies from various assembly strategies and sequencing technologies including Illumina short reads, 10× genomics linked-reads, PacBio and Oxford Nanopore long reads, and Bionano optical maps. We also discuss the PAV polymorphism of seven Tasmanian devil whole genome assemblies of normal animal tissues and devil facial tumour 1 (DFT1) and 2 (DFT2) samples, and the identification of bacterial sequences as contamination in some of the tumorous assemblies.

**Availability and implementation:**

The pipeline is available under the MIT License at https://github.com/wtsi-hpag/scanPAV.

**Supplementary information:**

Supplementary data are available at *Bioinformatics* online.

## 1 Introduction

For a complete catalogue of genetic variations, it is important to include Presence–Absence Variations (PAVs) as sources of genetic divergence and diversity together with SNPs/indels and CNVs. In this note, we present scanPAV, a novel algorithm for pairwise genome comparison that identifies and extracts sequences present in one assembly but absent in the other (PAVs). The identification of PAVs in genome comparisons can be useful to detect real polymorphism or lateral transfer, but can also help assess an assembly completeness, and strengths and weaknesses of a new technology or a new assembly pipeline.

Here, we present both types of PAV analyses using scanPAV: (i) the study of presence–absence sequences for the human reference GRCh38 and six other human assemblies, to assess the technologies used and the assembly strategies; and (ii) the PAVs detection for seven Tasmanian devil *de novo* assemblies from normal animal tissues and devil facial tumour samples (Stammnitz, M.R. *et al.*, The origins and vulnerabilities of two transmissible cancers in Tasmanian devils, in press at Cancer Cell).

## 2 Materials and methods

**Fig. 1.**
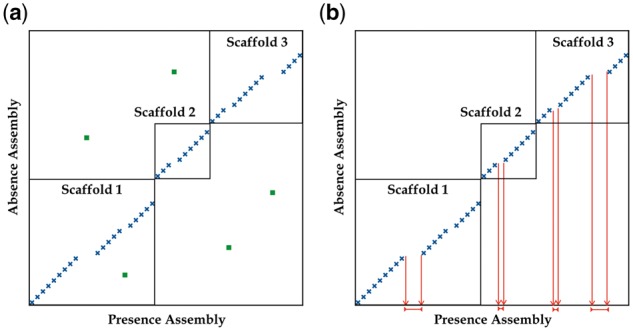
The scanPAV algorithm. (a) Presence assembly sequences are shred into 1 Kb fragments and mapped against the absence assembly: crosses are alignments consistent with the reference while squares are repeats; (b) small repeats [squares in (a)] are considered noise and filtered out; then uncovered regions in the presence assembly are identified as PAV sequences and printed out

When comparing two genome assemblies A and B, the scanPAV pipeline looks for sequences present in A (presence-assembly) but absent in B (absence-assembly). Figure 1 schematically shows the algorithm implemented in the pipeline. The fundamental steps performed are: (i) the presence-assembly is shred into 1 Kb fragments after the removal of N-bases; (ii) the obtained fragments are mapped against the absence-assembly using BWA (Li, 2013); then (iii) the alignments are processed to filter out small repeats and to identify the mapping coordinates (see Supplementary Note). Finally, (iv) the 1 Kb fragments from the presence-assembly missing in the absence-assembly are extracted and the adjacent ones are merged into a single sequence. All the presence PAVs are written into a fasta file and their details are collected into a VCF file. When running ScanPAV three parameters can be set: the aligner to be used (-align), the choice being between bwa or smalt (default: bwa); the number of threads for the aligner to use (-nodes, default: 20) and the minimum Smith-Waterman score for the alignments (-score, default: 550). Evaluation on the sensitivity and accuracy of the pipeline has been performed by adding simulated long indels on the C.elegans and the Human genome reference. For the human genome, ScanPAV was able to identify the 99% of the simulated long insertions and about 93% of the simulated long deletions, while 7% (1%) of the deletions (insertions) were missing from PAVs. More details in the Supplementary Note.

## 3 Results

Here, we show the use of scanPAV to assess the completeness of six human genome assemblies compared to the reference GRCh38. The assemblies tested are based on Sanger (HuRef) (Levy *et al.*, 2007), Illumina (Illumina) (Mostovoy *et al.*, 2016), Illumina plus Hi-C links (Hs2-HiC) (Dudchenko *et al.*, 2017), PacBio (AK1) (Seo *et al.*, 2016) and Oxford Nanopore data (ONT_30× and ONT_35×) (Jain *et al.*, 2018). The details of these assemblies are described in Supplementary Note. Supplementary Table S1 shows that all the scaffolded assemblies are very contiguous with N50s from tens to hundreds of Mbs. In most cases though the continuity is broken at contig level, where, except the reference, only the long-reads based AK1 and the ONT assemblies have N50 in the Mb range.

Three of the assemblies (GRCh38, HuRef and AK1) include a Y chromosome. Here, for a fairer comparison we compare PAVs after removal of chrY (see Supplementary Note for details). The scanPAV analysis results excluding chrY are shown in Table 1 for all possible pairs of assemblies. In this table, each row shows the length of PAVs of the presence-assembly (first column) that are missing in the other assemblies. In particular the first row gives the total length of sequences from the reference GRCh38 that are missing in the other assemblies. From the comparison with the reference GRCh38 it is clear that the most complete assemblies are AK1 and Hs2-HiC, which miss about 0.8% of the reference. They are closely followed by HuRef and the ONT assemblies, missing from 1.5% to 2% sequences of the reference. The most incomplete assembly appears to be the Illumina one: even though the assembly reaches an impressive 33 Mb scaffold N50, it is missing about 14% of the reference. This is perhaps not surprising because, compared to the other assemblies, the Illumina one includes the highest percentage of Ns (10% of the total number of bases), and in addition it has the shortest contig N50 (0.01 Mb, see Supplementary Note).

**Table 1.** PAVs sequences for the human assemblies: in each row the total length of sequences from the presence assembly (first column) missing in the other assemblies

PAV present in:	PAVs (Mb) absent in:						
	GRCh38	HuRef	AK1	Hs2-HiC	Illumina	ONT_30×	ONT_35×
GRCh38	0	46	23	25	438	63	47
HuRef	25	0	34	32	290	72	65
AK1	19	49	0	30	318	60	53
Hs2-HiC	27	50	37	0	284	74	64
Illumina	148	170	152	149	0	180	177
ONT_30	25	56	32	37	290	0	46
ONT_35	69	107	72	90	379	91	0

It is interesting to notice that the ONT contig N50 doubles its size when increasing the read depth with 5× ultra long reads and this also results in less missing sequences. But at 35× there are more ONT_35× sequences missing in GRCh38. These sequences failed to be mapped by scanPAV as they are heavily affected by the common nanopore sequencing error of homo-polymer shortening and deletions, indeed, their homo-polymer frequencies is up to 50% lower than that of the total ONT assembly.

In the Supplementary Note we discuss an additional structure assessment for some of these assemblies and we list the mis-joints we identified in HuRef, Hs2-HiC, AK1 and ONT_35x×.

The Tasmanian devil is affected by two distinct transmissible cancers which are endangering the devil population survival. In an attempt to understand how these cancers emerged and if there are viable treatments, six *de novo* assemblies have been generated for two healthy and four tumour samples (Stammnitz *et al*., 2018). A scanPAV analysis of these assemblies and the foreign DNA contamination it helped finding are discussed in the Supplementary Note.

## 4 Conclusion

Assessing the quality and completeness of a newly generated assembly is of the foremost importance in understanding the strength and weakness for new sequencing technologies and assembly algorithms. With scanPAV, it is possible to easily identify and isolate sequences present in one assembly but absent in another, providing a quantitative estimate of the completeness of an assembly. The scanPAV pipeline can also be useful in identifying real polymorphism and DNA sequencing library contamination.

## Supplementary Material

Supplementary Data
