# Supplementary material for: scanPAV: a pipeline for extracting presence–absence variations in genome pairs
Source: Bioinformatics. 2018 Mar 28;34(17):3022–4. doi: 10.1093/bioinformatics/bty189 (PMC6129304; doi:10.1093/bioinformatics/bty189)
Supplement: Supplementary Data [file bty189_supplementarynote.pdf]

Genome analysis

scanPAV: a pipeline for extracting presence-absence variations in genome pairs. Supplementary Note

Francesca Giordano [1,*], Maximilian R. Stammnitz [2], Elizabeth P. Murchison [2], and Zemin Ning [1,*]

[1]The Wellcome Sanger Institute, Wellcome Genome Campus, Hinxton, Cambridge, UK
[2]Transmissible Cancer Group, Department of Veterinary Medicine, University of Cambridge, UK

*To whom correspondence should be addressed

S1 Resources needed to run ScanPAV

The scanPAV pipeline, shown schematically in Figure S1, is the first tool which is capable of systematically screening PAVs. To scan two human sized genomes, it only takes about 60 CPU hours with a maximum RAM usage lower than 10 GB.

S2 Filtering of small repeats

For mapping purposes, the presence assembly is shredded into 1Kb fragments which are then aligned against the absence assembly. This “shred-and-align”strategy ensures an end-to-end match for a whole scaffold alignment. In some cases though, a 1Kb fragment can map to multiple locations in the absence assembly if it is a repetitive sequence. These cases are easy to spot in the alignment file as bwa will give them a low mapping score. Sometimes, one (or more) of this short repeat is within a longer sequence which does not map anywhere in the absence genome. In these cases, in the alignment we will see that most of the fragments from the scaffold under consideration will map consistently to a scaffold in the absence assembly, while a few of the fragments do not map anywhere. In between the not-mapping fragments, there might be one of more of the short repeats, that instead of mapping to the main absence-assembly scaffold, map somewhere else completely. These short repeats are generally characterised by low mapping score.

An example is given in Figure S2: most fragments for the scaffold under consideration map to the absence scaffold absence_1. The fragments presence_scaffold1_X011852000, presence_scaffold1_X011854000, presence_scaffold1_X011856000 and presence_scaffold1_X011858000, presence_scaffold1_X011859000, presence_scaffold1_X011860000 do not map anywhere, but they are interspersed by the three sequences, presence_scaffold1_X011853000 presence_scaffold1_X011855000 and presence_scaffold1_X011857000, which map partially and with low score (7, 48 and 0) to different scaffolds (absence_0, absence_2 and absence_0, respectively).

By not filtering these mappings out, scanPAV would print out 4 separate PAVs: 1) presence_scaffold1_X011852000, 2) presence_scaffold1_X011854000, 3) presence_scaffold1_X011856000, and 4) the concatenation of presence_scaffold1_X011858000, presence_scaffold1_X011859000 and presence_scaffold1_X011860000.

Instead, we believe the interspersed mappings to other scaffolds and with low mapping score are in truth noisy mapping, and let scanPAV filter these mappings out, so that all the fragments from presence_scaffold1_X011852000 to presence_scaffold1_X011860000 are concatenated and printed out as a unique long PAV.

S3 ScanPAV sensitivity test

We tested scanPAV sensitivity by randomly modifying a genome reference adding long indels with the package simulatesv (`https://github.com/mlliou112/simulatesv`). To incorporate some noise we also let simulatesv introduce SNPs with a frequency of 1 every 1000 bases. Then we used scanPAV to compare the modified and the original assemblies and extract absence (deletions) and presence (insertions) PAVs in the modified assembly. This test was performed on both the C.elegans and the human reference genomes (GRCh38).

C.elegans simulated PAVs

The pipeline simulatesv added 115 insertions for a total of 2.9 Mb and 101 deletions, a total of 2.4 Mb, in the C.elegans reference genome. ScanPAV recognised 123 absent PAVs and 128 presence PAVs. Of these, 13 absent (14 present) PAVs are 1000 bp long, and most of them (85%) are false positive. The same happened in the human case, therefore we recommend users to filter out any PAVs shorter or equal to 1000 bp as noise. We understand that some users might be interested in short PAVs, so scanPAV does not filter them automatically. Other than these short PAVs, no other false positive were found. ScanPAV identified all 101 deletions, but the sequence for some of them was split in two or more PAVs, and this is why there were a total of 109

© The Author 2015. Published by Oxford University Press. All rights reserved. For permissions, please e-mail: journals.permissions@oup.com

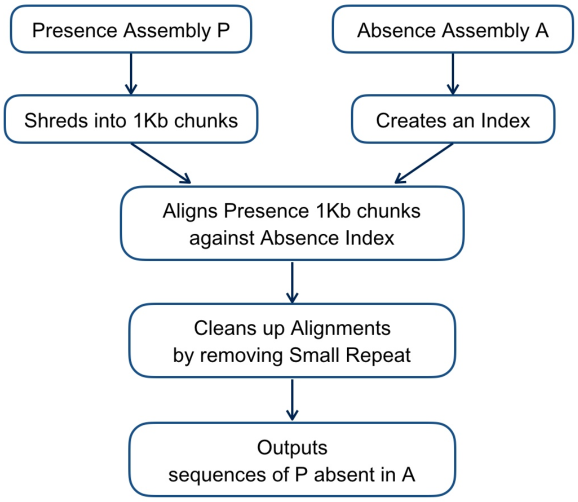

Fig. S1: The scanPAV pipeline.

absence PAVs. In addition, the sequences from two deletions were merged into one, as they were very close to each other in the modified assembly (about 900 bp). ScanPAV also recognised and outputted all 115 insertions.

Human simulated PAVs

For the human assembly, a total of 305 insertions (7.6 Mbp) and 291 deletions (7.4 Mbp) were added to the GRCh38 assembly. ScanPAV identified a total of 429 absence PAVs and 441 presence PAVs. As for the C.elegans, the PAVs equal to 1000 bp were mostly false positives (98%). There were 304 absence and 304 presence PAVs longer than 1000 bp. Of these PAVs, one absence and one presence sequences were false positive. In both cases these were sequences shorter than 2000 bp in between long N-gaps, which confused the mapping.

ScanPAV identified correctly 271 deletions (93%) although, as for the C.elegans, the sequences for some of the deletions were split in multiple absence PAVs; 20 deletions (7%) were completely missed by scanPAV, likely because their sequences were repeatitive and mapped to some other genome region. ScanPAV then identified correctly 303 (99%) of the insertions, while two insertions (1%) were missing.

S4 Description of human genome assemblies analysed with scanPAV

In our application note we presented the scanPAV analysis for six human genome assembly and the GRCh38 reference. Here, we describe in a bit more details how the six assemblies have been generated and the platforms used.

1. The HuRef assembly (Levy *et al.*, 2007) was published in 2007. It has been generated by the Celera pipeline (Myers *et al.*, 2000) using about 7.5x depth of whole-genome shotgun Sanger sequencing data (800 bp paired-end reads) mostly from a single Caucasian male individual;
2. The Illumina assembly (Mostovoy *et al.*, 2016) is based on 39x short-insert size and 24x long-insert size Illumina 101 bp paired-end reads from the NA12878 cell line. The assembly has been generated by spaDES (Nurk *et al.*, 2013), and then scaffolded using 10x genomics linked-reads and Bionano optical maps;
3. Hs2-HiC (Dudchenko *et al.*, 2017) is based on a DISCOVAR *de novo* (Love *et al.*, 2013) draft assembly of 60x Illumina 250 bp paired-end reads from the NA12878 cell line. The draft scaffolds have been then ordered, oriented and joint together by the authors using the Hi-C data, generating an assembly with about 91% of the sequences in 23 large scaffolds that correspond to the human 23 chromosomes.

We also analysed three assemblies based on the long read technologies of PacBio and Oxford Nanopore (ONT).

4. The PacBio-based assembly AK1 (Seo *et al.*, 2016) is initially generated by Falcon using 101x PacBio reads with a mean length of 10 Kb and then polished with Quiver. A first round of base errors has been performed by Pilon using Illumina short reads. It is then scaffolded twice with two independent Bionano genome maps and finally polished again with Pilon. AK1 is the most continuous and accurate assembly between the ones analysed in our application note.
5. In Jain *et al.*, 2017 the authors show for the first time a number of complete human assemblies (NA12878 cell line) based on Oxford Nanopore data. We focused on two of these assemblies both generated by Canu (Koren *et al.*, 2017): (1) ONT_30x, based on 30x ONT long reads (mean length 11 Kb), and (2) ONT_35x, based on the same 30x reads plus 5x of ultra-long reads with read N50 65 Kb, and with the longest fully mapped read 882 Kb long. Both assemblies base errors have been corrected by three (two) rounds of Pilon using short Illumina reads, respectively. Although Nanopore read accuracies are generally lower than other technologies, after the polishing steps with illumina data the average identity reached is about 99.3% with respect to the GRCh38 reference. The scanPAV analysis revealed that these assemblies are both quite complete, in addition, some of the missing PAVs could be recoverable as they are present in the assemblies but not recognised because of remaining base errors, in particular deletions in homo-polymers stretches.

Table S1. Statistic information for the Human assemblies analysed in the application note.

Assembly	Bases (Gb)	#Contigs (#Scaffolds)	Longest (Mb)	Contig-n50 (Mb)	Scaffold-n50 (Mb)
GRCh38	3.1	24	249	59	156
HuRef	2.8	3,134	235	0.11	144
Illumina	2.9	170	100	0.01	33
Hs2-HiC	2.8	44,065	225	0.10	141
AK1	2.9	2,832	114	18	45
ONT_30x	2.8	2,886	28	4	–
ONT_35x	2.9	2,337	50	8	–

Table S2. PAVs sequences for the human assemblies from male individuals including chromosome Y: in each row the total length of sequences from the presence assembly (first column) missing in the other assemblies.

PAV present in:	PAVs (Mb) Absent in: GRCh38	HuRef	AK1
GRCh38	0	42	24
HuRef	26	0	35
AK1	21	46	0

S5 ScanPAV analysis for the human assemblies from male individuals

Three of the assemblies tested (GRCh38, HuRef and AK1) include a Y chromosome. For a fairer comparison of missing sequences from all six assemblies, in the application note we ignored PAVs from chrY. To do this, before our PAV analysis we took out chrY from the GRCh38 assembly. For HuRef and AK1 instead, we took out all of the scaffolds that mapped to chrY but did not map significantly to chrX, to account for the pseudoautosomal regions, common to chrX and chrY. For these assemblies from male individuals we report in Table S2 a separate PAV analysis that includes chrY and all the scaffolds related to it.

S6 Inter-chromosomal mis-joint analysis of the human assemblies

As discussed in the main note, the AK1 assembly is the most complete one according to the scanPAV analysis, and it misses only 0.8% of the GRCh38 assembly, but is is very closely followed by Hs2-HiC, missing only 0.9% of the reference. Next, HuRef and the ONT assemblies miss from 1.6% to 2% of GRCh38. The Illumina assembly instead misses about 12% of the reference. Extracting and estimating the size of the missing sequences in a newly generated assembly is not enough though to evaluate the assembly quality. In addition to estimating PAVs, we also performed a mis-joint analysis on some of the most complete assemblies: AK1, HuRef, Hs2-HiC and the higher depth ONT assembly, ONT_35x.

For the mis-joint analysis, we looked for scaffolds that map to multiple chromosomes. For the AK1 and ONT_35x we generated chromosome-level scaffolds based on synteny with GRCh38, (called "pseudo_chr" in the following): (1) the original scaffolds (or contigs) have been mapped to GRCh38 using Minimap2 (Li, 2017) (2) if a scaffold mapped to a single chromosome, the scaffold has been assigned to that chromosome; otherwise, the scaffold has been assigned to the chromosome with the longest alignment; (3) the order of the scaffolds (contigs) in the chromosome-level scaffolds have been inferred from their relative mapping to the reference; (4) if the aligner could not assign a scaffold to any chromosome, the scaffold is unassigned and ignored in our mis-joint analysis. For the ONT_35x, 379 contigs were unplaced with about 18M bases, or 0.7% of the initial assembly; for the AK1, 787 scaffolds were unplaced with about 21M bases, or again about the 0.7% of the assembly. The assemblies HuRef and Hs2-HiC already provide chromosome-level scaffolds: we analysed these ignoring the smaller unassigned scaffolds. Because of the large number of similar repeats in the X and Y chromosomes in the GRCh38 reference that could be misinterpreted as mis-joints, we did not include chromosome Y in our estimation of mis-joints.

For each assembly, we mapped the chromosome-level scaffolds to the reference, and confirmed that they majorly mapped to a single reference chromosome. We filtered out all alignments with an average identity smaller than 70% and the alignment with mapping score smaller than 10, to exclude repeats. If there were blocks of alignments of at least 300 Kb mapping to a chromosome different from the major one, we considered them mis-joints. For mapping the scaffolds we tested as aligner both Minimap2 (Li, 2017) and SMALT (Ponstingl, H. and Ning, Z., SMALT: a mapper for DNA sequencing reads `http://www.sanger.ac.uk/science/tools/smalt-0`). Minimap2 tends to find longer blocks with lower average identity, which translates into few more mis-joint blocks. This suggests that the reported length of the misplaced blocks are to be considered only as approximations, and a more in depth analysis should be performed to identify the exact location of the breaking points. Here we are reporting only the mis-joint blocks identified by both aligners.

The results of this mis-joint analysis for the four assemblies are shown in table S3 and are visualised in figure S3. Our pipeline did not find any inter-chromosomal mis-joint in the HuRef assembly. For the Hs2-HiC assembly, we found two misplaced blocks: a smaller one in Hs2-HiC_hic_scaffold_9 (310 Kb) and a larger one in Hs2-HiC_hic_scaffold_10 (1.2 Mb). For the AK1 assembly our pipeline indicated the presence of a large block of 16 Mb in the pseudo_chr16 that mapped to chr2 (instead of chr16) with an identity > 99.7%. As the chromosome-level scaffolds have been created by us according to synteny with GRCh38 for AK1, we looked into the original scaffolds and found the one that originated the mis-joint, KV784727.1, as shown in table S4. The ONT_35x assembly had by far the most mis-joints: 15 misplaced blocks were found. In particular we found a very long mis-joint block of 14 Mb and ten long blocks with length between 1 and 6 Mb. The locations of the mis-joints in the original contigs are reported in table S4.

S7 PAVs for seven Tasmanian devil assemblies

The scanPAV pipeline was used to study presence/absence variations in six newly generated assemblies from healthy and tumour Tasmanian devil samples. The Tasmanian devil is affected by two distinct transmissible cancers which are endangering the devil population survival (Pearse *et al.*, 2006; Pye *et al.*, 2016). In an attempt to understand how these cancers emerged and if there are viable treatments, six *de novo* assemblies have been generated for two healthy (202H1, 203H) and four tumour samples: two DFT2 tumour samples 202T2, 203T3, and two DFT1 tumour samples 86T and 88T, all derived from cell lines (Stammnitz, M.R. *et al.*, The origins and vulnerabilities of two transmissible cancers in Tasmanian devils, in press at Cancer Cell). Using scanPAV, we extracted PAV sequences for each possible pair of the six assemblies plus the Tasmanian devil references Ref-v7.1 (Murchison *et al.*, 2012) and PSU (Miller *et al.*, 2011). Statistic metrics for these assemblies are shown in Table S5. The lengths of the extracted PAVs are shown in Table S6.

The absent PAVs are significantly longer for the reference Ref-v7.1 and the PSU assembly compared to the more recently generated assemblies. There are some real polymorphisms between the samples, however, the more recent assemblies appear to be more complete than the two references. This could be due to improvement in the assembly strategy in time, for instance, that lead to a better scaffold- and contig-n50s, as shown in Table S5). In particular, even though the two references are longer (about 3.2 Gb), they also have an higher N content, and the number of bases after N removals is smaller than the one for the newer assemblies after N removal (see "Contig Bases" in Table S5).

The PAVs present in the tumour samples (202T2, 203T3, 86T and 88T) but absent in the reference Ref-v7.1 were screened against the NCBI database, to determine if the samples contain viruses or bacteria. The only sequences of non-devil origin revealed by the screening belong to two laboratory cell culture contaminants: *Mycoplasma arginini* and *Streptococcus pneumoniae*. No traces of foreign DNA sequences were found in the two references Ref-v7.1 and PSU, suggesting that the DNA samples sequenced were free of contamination (Stammnitz, M.R. *et al*, in press at Cancer Cell).

S8 Data Availability

All the PAV sequences extracted for the human and the Tasmanian devil assemblies, and the sensitivity test results using simulated long indels on the C.elegans and the human genomes are freely available online: ftp://ftp.sanger.ac.uk/pub/users/zn1/scanPAV.

Table S3. Inter-chromosomal mis-joints for each human assembly. For AK1 and ONT_35x the mis-joint pipeline was applied to chromosome assigned contigs from synteny with the reference, as described in section S6.

Assembly	Mis-Joint	Scaffold	Major Chromosome	Mis-Joint length (> 200 Kb) (bp)	Mapping Chromosome	Avg Id
HuRef	0	–	–	–	–	
Hs2-HiC	1	Hs2-HiC_hic_scaffold_9	chr19	300 K	chr22	99.0%
	2	Hs2-HiC_hic_scaffold_10	chr21	1.3 M	chr22	99.3%
AK1	1	pseudo_chr16	chr16	16 M	chr2	99.7%
ONT_35x	1	pseudo_chr1	chr1	3.9 M	chr19	99.8%
	2	pseudo_chr1	chr1	565 K	chr3	99.0%
	3	pseudo_chr2	chr2	1.8 M	chr1	99.1%
	4	pseudo_chr4	chr4	316 K	chr20	99.1%
	5	pseudo_chr4	chr4	543 K	chr5	99.2%
	6	pseudo_chr5	chr5	2.2 M	chr18	99.1%
	7	pseudo_chr6	chr6	3.3 M	chr7	99.1%
	8	pseudo_chr8	chr8	3.7 M	chr16	98.9%
	9	pseudo_chr10	chr10	5.8 M	chr1	99.0%
	10	pseudo_chr10	chr10	2.0 M	chr11	98.9%
	11	pseudo_chr12	chr12	13.9 M	chr10	99.1%
	12	pseudo_chr13	chr13	4.2 M	chr10	99.1%
	13	pseudo_chr15	chr15	2.0 M	chr3	98.8%
	14	pseudo_chr16	chr16	1.4 M	chr1	98.8%
	15	pseudo_chr22	chr22	342 K	chr19	98.6%

References

Dudchenko, O. *et al.*, (2017), *De novo* assembly of the Aedes aegypti genome using Hi-C yields chromosome-length scaffolds, *Science*, doi:10.1126/science.aal3327.
Jain, M. *et al.*, (2017), Nanopore sequencing and assembly of a human genome with ultra-long reads, *bioRxiv*, doi:10.1101/128835.
Koren, S. *et al.*, (2017), Canu: scalable and accurate long-read assembly via adaptive k-mer weighting and repeat separation, *Genome Research* doi:10.1101/gr.215087.116.
Levy, S. *et al.*, (2007), The Diploid Genome Sequence of an Individual Human, *PLOS Biology* **5(10)**, e254.
Li, H., (2017), Minimap2: fast pairwise alignment for long nucleotide sequences, *arXiv*, **1708.01492**.
Love, R. R. *et al.*, (2016), Evaluation of DISCOVAR *de novo* using a mosquito sample for cost-effective short-read genome assembly, *BMC Genomics* **17**, 187.
Miller, W. *et al.*, (2011), Genetic diversity and population structure of the endangered marsupial Sarcophilus harrisii (Tasmanian devil). *PNAS*, **108**, 12348.
Mostovoy, Y. *et al.*, (2016), A hybrid approach for *de novo* human genome sequence assembly and phasing, *Nature Methods*, **13**, 587.
Murchison, E. P. *et al.*, (2012), Genome sequencing and analysis of the Tasmanian devil and its transmissible cancer, *Cell*, **148**, 780.

Table S4. Mis-joint locations in the original scaffolds for assemblies AK1 and ONT_35x.

Assembly	Mis-Joint	Original Scaffold	1st Chromosome	Length mapped to 1st Chromosome	Break Position	2nd Chromosome	Length mapped to 2nd Chromosome
AK1	1	KV784727.1	chr16	16.9 M	16,390,000 – 16,390,001	chr2	16.3 M
	1	tig00001490_pilon_pilon	chr1	5.5 M	3,999,036 – 4,000,001	chr19	3.9 M
	2	tig01414718_pilon_pilon	chr1	23.6 M	23,710,000 – 23,713,101	chr3	561 K
	3	tig00000928_pilon_pilon	chr1	2.0 M	2,108,108 – 2,110,001	chr2	3.0 M
	4	tig00000326_pilon_pilon	chr4	10.2 M	330,000 – 365,771	chr20	322 K
	5	tig01414909_pilon_pilon	chr4	9.5 M	557,558 – 560,001	chr5	535 K
	6	tig01415181_pilon_pilon	chr5	9.2 M	9,740,000 – 9,741,384	chr18	2.2 M
	7	tig00000726_pilon_pilon	chr6	6.9 M	3,399,916 – 3,400,054	chr7	3.4 M
ONT_35x	8	tig00001250_pilon_pilon	chr8	4.7 M	4,820,000 – 4,820,001	chr16	3.7 M
	9	tig01414799_pilon_pilon	chr10	14.4 M	14,700,000 – 14,770,556	chr1	5.8 M
	10	tig01415009_pilon_pilon	chr10	9.1 M	2,037,882 – 2,040,001	chr11	2.0 M
	11	tig01414760_pilon_pilon	chr12	18.0 M	18,455,260 – 18,460,001	chr10	13.9 M
	12	tig01414699_pilon_pilon	chr13	45.2 M	46,129,991 – 46,131,368	chr10	4.2 M
	13	tig00002429_pilon_pilon	chr15	7.3 M	7,426,592 – 7,430,001	chr3	2.0 M
	14	tig00001215_pilon_pilon	chr16	3.9 M	1,719,551 – 2,010,001	chr1	1.6 M
	15	tig00000735_pilon_pilon	chr22	11.0 M	358,160 – 360,002	chr19	352 K

Table S5. Statistic information for the Tasmanian devil assemblies.

Assembly	Bases (Gb)	#Scaffolds	Longest (Mb)	Scaffold-n50 (Mb)	Contig Bases (Gb)	Contig-n50 (Kb)
Ref-v7.1	3.2	35,974	5.3	1.8	2,9	20
PSU	3.2	148,774	2.9	0.15	2.9	11
202T2	3.0	61,915	50.7	9.5	3.0	55
202H1	3.0	67,871	22.0	4.2	3.0	51
203T3	3.0	62,742	50.7	9.6	3.0	53
203H	3.0	63,553	31.1	4.2	3.0	48
86T	3.0	70,410	24.5	4.4	3.0	66
88T	3.0	61,028	19.7	4.0	3.0	60

Table S6. PAVs sequences for the Tasmanian devil's assemblies: in each row the total length of sequences from the presence assembly (first column) missing in the other assemblies

PAV present in:	PAVs (Mb) Absent in: Ref-v7.1	PSU	202T2	202H1	203T3	203H	86T	88T
Ref-v7.1	0	203	83	89	83	85	77	79
PSU	158	0	94	101	95	97	88	91
202T	129	176	0	36	25	29	28	26
202H	124	171	28	0	30	27	28	28
203T	127	174	22	34	0	27	26	25
203H	127	173	28	33	30	0	28	27
86T	131	175	28	39	29	31	0	22
88T	129	173	27	35	28	27	23	0

Myers, E.W. *et al*., (2000), A whole-genome assembly of Drosophila, *Science* **287(5461)**, 2196.

Nurk, S. *et al*., (2013), Assembling Genomes and Mini-metagenomes from Highly Chimeric Reads, *Research in Computational Molecular Biology: 17th Annual International Conference, RECOMB 2013, Beijing, China, April 7-10, 2013. Proceedings*, 158.

Pearse, A.M., and Swift, K., (2006), Allograft theory: transmission of devil facial-tumour disease, *Nature*, **439**, 549.

Pye, R.J. *et al*, (2016). A second transmissible cancer in Tasmanian devils, *Proc Natl Acad Sci USA*, **113** 374.

Seo, J.S. *et al*., (2016), *De novo* assembly and phasing of a Korean human genome, *Nature*, **538**, 243.

Presence_Fragment	Start_Position	Mapped_Length	Absence_Scaffold	Absence_Start	Absence_End	Mapping_Score	Avg_Identity
presence_scaffold1_X011836000	11836000	1000	absence_1	11887603	11888602	59	100.00
presence_scaffold1_X011837000	11837000	1000	absence_1	11888603	11889602	57	100.00
presence_scaffold1_X011838000	11838000	1000	absence_1	11889603	11890602	57	100.00
presence_scaffold1_X011839000	11839000	1000	absence_1	11890603	11891602	58	100.00
presence_scaffold1_X011840000	11840000	1000	absence_1	11891603	11892602	57	100.00
presence_scaffold1_X011841000	11841000	1000	absence_1	11892603	11893602	55	100.00
presence_scaffold1_X011842000	11842000	1000	absence_1	11893603	11894602	55	100.00
presence_scaffold1_X011843000	11843000	1000	absence_1	11894603	11895602	58	100.00
presence_scaffold1_X011844000	11844000	1000	absence_1	11895603	11896602	55	100.00
presence_scaffold1_X011845000	11845000	1000	absence_1	11896603	11897602	56	100.00
presence_scaffold1_X011846000	11846000	1000	absence_1	11897603	11898602	51	100.00
presence_scaffold1_X011847000	11847000	1000	absence_1	11898603	11899602	57	100.00
presence_scaffold1_X011848000	11848000	1000	absence_1	11899603	11900602	57	100.00
presence_scaffold1_X011849000	11849000	1000	absence_1	11900603	11901602	55	100.00
presence_scaffold1_X011850000	11850000	1000	absence_1	11901603	11902602	57	100.00
presence_scaffold1_X011851000	11851000	148	absence_1	11902603	11902750	50	100.00
presence_scaffold1_X011852000	11852000	0	*	0	0	00	0.00
presence_scaffold1_X011853000	11853000	174	absence_0	2030847	2031223	07	84.47
presence_scaffold1_X011854000	11854000	0	*	0	0	00	0.00
presence_scaffold1_X011855000	11855000	135	absence_2	11992555	11992874	48	82.57
presence_scaffold1_X011856000	11856000	0	*	0	0	00	0.00
presence_scaffold1_X011857000	11857000	106	absence_0	1211276	1211414	00	92.09
presence_scaffold1_X011858000	11858000	0	*	0	0	00	0.00
presence_scaffold1_X011859000	11859000	0	*	0	0	00	0.00
presence_scaffold1_X011860000	11860000	0	*	0	0	00	0.00
presence_scaffold1_X011861000	11861000	719	absence_1	11902751	11903469	57	100.00
presence_scaffold1_X011862000	11862000	1000	absence_1	11903470	11904469	59	100.00
presence_scaffold1_X011863000	11863000	1000	absence_1	11904470	11905469	57	100.00
presence_scaffold1_X011864000	11864000	1000	absence_1	11905470	11906469	57	100.00
presence_scaffold1_X011865000	11865000	1000	absence_1	11906470	11907469	60	100.00
presence_scaffold1_X011866000	11866000	1000	absence_1	11907470	11908469	57	100.00
presence_scaffold1_X011867000	11867000	1000	absence_1	11908470	11909469	58	100.00
presence_scaffold1_X011868000	11868000	1000	absence_1	11909470	11910469	58	100.00
presence_scaffold1_X011869000	11869000	1000	absence_1	11910470	11911469	57	100.00
presence_scaffold1_X011870000	11870000	1000	absence_1	11911470	11912469	59	100.00
presence_scaffold1_X011871000	11871000	997	absence_1	11912470	11913469	58	99.90
presence_scaffold1_X011872000	11872000	1000	absence_1	11913470	11914469	60	100.00
presence_scaffold1_X011873000	11873000	1000	absence_1	11914470	11915469	57	100.00
presence_scaffold1_X011874000	11874000	1000	absence_1	11915470	11916469	57	100.00
presence_scaffold1_X011875000	11875000	1000	absence_1	11916470	11917469	58	100.00
presence_scaffold1_X011876000	11876000	1000	absence_1	11917470	11918469	57	100.00
presence_scaffold1_X011877000	11877000	997	absence_1	11918470	11919469	58	99.90
presence_scaffold1_X011878000	11878000	1000	absence_1	11919470	11920469	35	100.00

(a)

Presence_Fragment	Start_Position	Mapped_Length	Absence_Scaffold	Absence_Start	Absence_End	Mapping_Score	Avg_Identity
presence_scaffold1_X011836000	11836000	1000	absence_1	11887603	11888602	59	100.00
presence_scaffold1_X011837000	11837000	1000	absence_1	11888603	11889602	57	100.00
presence_scaffold1_X011838000	11838000	1000	absence_1	11889603	11890602	57	100.00
presence_scaffold1_X011839000	11839000	1000	absence_1	11890603	11891602	58	100.00
presence_scaffold1_X011840000	11840000	1000	absence_1	11891603	11892602	57	100.00
presence_scaffold1_X011841000	11841000	1000	absence_1	11892603	11893602	55	100.00
presence_scaffold1_X011842000	11842000	1000	absence_1	11893603	11894602	55	100.00
presence_scaffold1_X011843000	11843000	1000	absence_1	11894603	11895602	58	100.00
presence_scaffold1_X011844000	11844000	1000	absence_1	11895603	11896602	55	100.00
presence_scaffold1_X011845000	11845000	1000	absence_1	11896603	11897602	56	100.00
presence_scaffold1_X011846000	11846000	1000	absence_1	11897603	11898602	51	100.00
presence_scaffold1_X011847000	11847000	1000	absence_1	11898603	11899602	57	100.00
presence_scaffold1_X011848000	11848000	1000	absence_1	11899603	11900602	57	100.00
presence_scaffold1_X011849000	11849000	1000	absence_1	11900603	11901602	55	100.00
presence_scaffold1_X011850000	11850000	1000	absence_1	11901603	11902602	57	100.00
presence_scaffold1_X011851000	11851000	148	absence_1	11902603	11902750	50	100.00
presence_scaffold1_X011852000	11852000	0	*	0	0	00	0.00
presence_scaffold1_X011853000	11853000	0	*	0	0	00	0.00
presence_scaffold1_X011854000	11854000	0	*	0	0	00	0.00
presence_scaffold1_X011855000	11855000	0	*	0	0	00	0.00
presence_scaffold1_X011856000	11856000	0	*	0	0	00	0.00
presence_scaffold1_X011857000	11857000	0	*	0	0	00	0.00
presence_scaffold1_X011858000	11858000	0	*	0	0	00	0.00
presence_scaffold1_X011859000	11859000	0	*	0	0	00	0.00
presence_scaffold1_X011860000	11860000	0	*	0	0	00	0.00
presence_scaffold1_X011861000	11861000	719	absence_1	11902751	11903469	57	100.00
presence_scaffold1_X011862000	11862000	1000	absence_1	11903470	11904469	59	100.00
presence_scaffold1_X011863000	11863000	1000	absence_1	11904470	11905469	57	100.00
presence_scaffold1_X011864000	11864000	1000	absence_1	11905470	11906469	57	100.00
presence_scaffold1_X011865000	11865000	1000	absence_1	11906470	11907469	60	100.00
presence_scaffold1_X011866000	11866000	1000	absence_1	11907470	11908469	57	100.00
presence_scaffold1_X011867000	11867000	1000	absence_1	11908470	11909469	58	100.00
presence_scaffold1_X011868000	11868000	1000	absence_1	11909470	11910469	58	100.00
presence_scaffold1_X011869000	11869000	1000	absence_1	11910470	11911469	57	100.00
presence_scaffold1_X011870000	11870000	1000	absence_1	11911470	11912469	59	100.00
presence_scaffold1_X011871000	11871000	997	absence_1	11912470	11913469	58	99.90
presence_scaffold1_X011872000	11872000	1000	absence_1	11913470	11914469	60	100.00
presence_scaffold1_X011873000	11873000	1000	absence_1	11914470	11915469	57	100.00
presence_scaffold1_X011874000	11874000	1000	absence_1	11915470	11916469	57	100.00
presence_scaffold1_X011875000	11875000	1000	absence_1	11916470	11917469	58	100.00
presence_scaffold1_X011876000	11876000	1000	absence_1	11917470	11918469	57	100.00
presence_scaffold1_X011877000	11877000	997	absence_1	11918470	11919469	58	99.90
presence_scaffold1_X011878000	11878000	1000	absence_1	11919470	11920469	35	100.00

(b)

Fig. S2: Short repeats filtering: an example. (a) Some 1Kb fragments within a PAV sometimes map to different scaffolds compared to the majority of the other fragments. Their mappings are characterised by low mapping score and scanPAV considers them noise. (b) These short repeat mappings are filtered out and a long sequence that concatenates all the fragments from presence_scaffold1_X011852000 to presence_scaffold1_X011860000 is extracted as a single PAV.

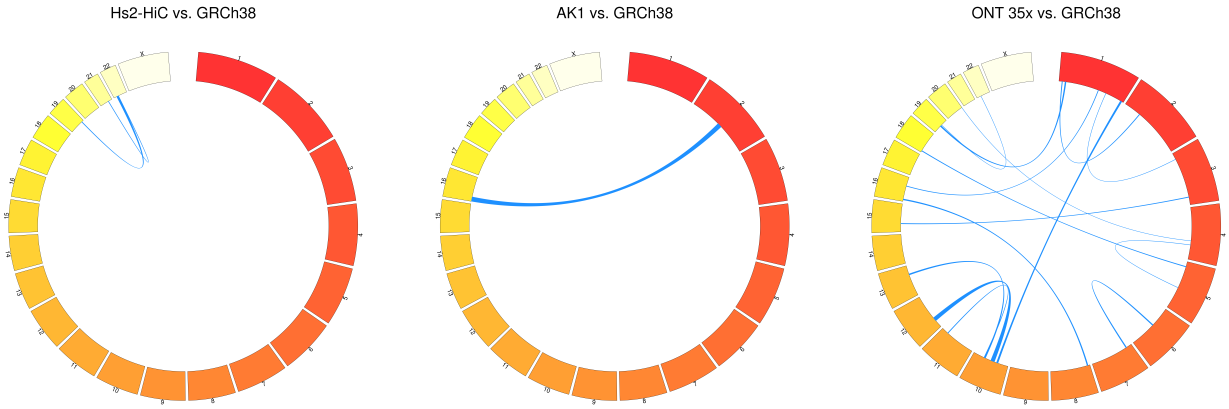

Fig. S3: Misplaced block visualisation for chromosome assigned scaffolds in Hs2-HiC (left), AK1 (center) and ONT_35x (right).
